# Conjugable, Antifouling, and Non-immunogenic Coatings for Gold Nanoparticles by Multivalent Grafting of Azide-Bearing Polyoxazoline Brushes

**DOI:** 10.1021/acs.langmuir.5c04094

**Published:** 2026-01-28

**Authors:** Tobias Komsthöft, Michele do Nascimento Tomaz, Pedro R. M. Veloso, Ana R. S. Ribeiro, Lucca Trachsel, Jutta Horejs-Höck, Mark W. Tibbitt, Samuele Tosatti, Emanuele Papini, Stefan Zürcher, Fabrizio Mancin

**Affiliations:** † SuSoS AG, Lagerstrasse 14, 8400 Dübendorf, Switzerland; ‡ Macromolecular Engineering Laboratory, Department of Mechanical and Process Engineering, ETH Zurich, 8092 Zurich, Switzerland; § Department of Chemical Sciences, University of Padova, Via F. Marzolo 1, 35121 Padova, Italy; ∥ Department of Biomedical Sciences, University of Padova, Via U. Bassi 58/b, 35121 Padova, Italy; ⊥ Department of Biosciences and Medical Biology, Paris Lodron University of Salzburg, 5020 Salzburg, Austria; # Department of Chemistry, 6612 Carnegie Mellon University, 4400 Fifth Avenue, Pittsburgh, Pennsylvania 15213, United States

## Abstract

Inorganic nanoparticles (NP), particularly gold nanoparticles, hold great promise for biomedical applications. However, one of the major challenges in the implementation of therapeutic applications based on inorganic nanoparticles is the individuation of appropriate coatings, which are indeed responsible for stability, escape from the immune system, and further functionalization. In this paper, we explore the properties of the multi-azide-containing and highly nonfouling polymer PAcrAm-*g*-(PMCA, NH2, ND), based on poly­(oxazoline) derivatives grafted to a poly­(acrylamide), as a coating for gold nanoparticles that can grant stability and easiness of derivatization and can prevent nonspecific protein adhesion on gold nanoparticles (Au-NP). We found that the addition of the polymer to citrate-stabilized gold nanoparticles led to the formation of NP suspensions with excellent colloidal stability, as confirmed by dynamic light scattering (DLS), ζ potential, thermogravimetric analysis (TGA), and transmission electron microscopy (TEM) investigations. In addition, the AuNP coating strongly decreases the protein adsorption corona and does not activate dendritic cells (DCs), showing low immunogenicity and potentially decreasing the NP clearance by the mononuclear phagocyte system.

## Graphical abstract

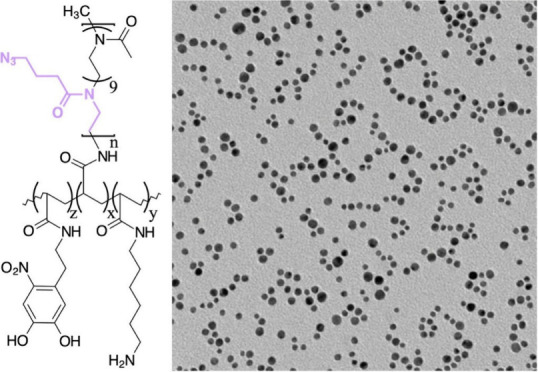

## Introduction

Inorganic nanoparticles (NP) have found a variety of potential applications in biomedicine, including in therapeutics, diagnostics, and biosensors and as building blocks for new materials. The interest toward this class of nanoparticles is justified by their peculiarities with respect to their organic counterpart, i.e., liposomes, lipid nanoparticles, and polymer nanoparticles. These peculiarities include the possibility of obtaining well-defined shapes and structures and the unique properties that several inorganic materials acquire when reduced to the nanoscale, such as superparamagnetism, surface plasmon resonance, and quantum confinement effects. Among the different materials, gold nanoparticles (Au-NP) offer many advantages such as reproducible synthesis, controllable size, and strong surface plasmon resonance.^1,2^

However, in most if not all of the applications of NP, the term inorganic is seldom fully correct, since they must be coated with organic molecules, polymers, or biomolecules to grant them colloidal stability or additional functionalization.^3–7^ Additionally, when the envisaged NP application is in the biomedical field, NP must be designed to avoid clearance by the mononuclear phagocyte system. This process, which removes NP from circulation, is usually initiated by the adhesion of opsonins on the surface of the NP, which tags them for elimination by phagocytes.^8–12^ Opsonization not only facilitates the uptake of NP by macrophages but also can activate the complement cascade, triggering an inflammatory response.^13–16^ Again, the most common strategy for overcoming this challenge is coating the NP with hydrophilic, nonfouling polymers, like poly­(ethylene glycol) (PEG), that suppress the nonspecific binding of serum proteins, including opsonins, decreasing the clearance of the NP by the immune system.^3,8,9,17^

While PEG is still considered the gold standard for these nonfouling coatings, disadvantages like oxidation and the potential formation of anti-PEG antibodies, due to the prolonged exposure to PEG that occurs to many individuals through detergents, cosmetics, and food, have motivated the research community to investigate alternative nonfouling polymers.^18–21^ These include polyoxazolines [in particular poly­(2-methyl-2-oxazoline) (PMOXA)], which are easy to synthesize and in selected cases are more biocompatible, hydrophilic, stable, and have a lower viscosity than PEG.^9,19,22–28^ Such features are mainly due to their tertiary amide backbone that ensures stability against hydrolysis, oxidation, and environmental degradation.^29^ These features, coupled with the high water solubility of PMOXA, have attracted strong interest for developing polyoxazoline-based antifouling coatings capable of enduring physiological conditions for extended periods compared to PEG analogues.^30^ However, also in the case of polyoxazolines, antifouling properties cannot be sufficient to ensure biocompatibility. Indeed, some of us recently demonstrated that complement and innate immune system proteins (such as C3 and ficolin FNC2) can selectively target PMOXA, triggering capture by macrophages and monocytes.^31,32^

Besides suppressing nonspecific fouling, an ideal nanoparticle-based system for biomedical applications should also allow the binding of different molecules on its surface, either for active targeting or to carry bioactive molecules, including drugs. A very popular method for achieving bioconjugation of polymers is azide–alkyne chemistry.^33–37^ Bioorthogonal reactions, which include azide–alkyne chemistry, are indeed a versatile strategy for polymer modification without cross-reactions or generation of byproducts.^38–40^ This allows cell targeting, therapeutic, and diagnostic characteristics to be imparted to the NP surface.^41^ Previously, we confirmed that the multifunctional, azide-containing, and nonfouling polyoxazoline polymers PAcrAm-*g*-(PMCA, NH2, ND), where PMCA is poly­(2-methyl-2-oxazoline-*co*-2-(3-azidopropyl)-2-oxazoline), significantly suppress the nonspecific adhesion of proteins on flat gold surfaces, while simultaneously showing a high capacity for bioorthogonal strain-promoted azide–alkyne cycloaddition (SPAAC) for further modification.^42^

Here, we demonstrate that this nonfouling and multiazide polymer can coat and stabilize Au-NP, granting them prolonged shelf life, decreased nonspecific and complement protein adhesion, and the potential for further modification via azide–alkyne coupling thanks to the azide groups. Additionally, the biocompatibility and, most importantly, the low immunogenicity of these nanoparticles were further confirmed by the absence of any activation of primary dendritic cells in vitro.

## Experimental Section

### General

Silver nitrate (AgNO_3_), sodium citrate dihydrate, gold­(III) chloride trihydrate, *N*-(2-hydroxyethyl)­piperazine-*N*′-(2-ethanesulfonic acid) (HEPES), methanol, Coomassie brilliant blue, β-mercaptomethanol, phosphate-buffered saline (10-fold concentrated), acetic acid, and sodium dodecyl sulfate (SDS) were purchased from Merck. CD86-FITC, HLA-DR-BV510, PD-L1-PE-Cy7, and CD1c-BV421 were purchased from BD Biosciences. Fixable viability dye eFluor 780 was purchased from Thermo Fisher Scientific.

### Polymer Synthesis

PAcrAm-*g*-(X, NH2, ND) samples (X = PMCA or PMOXA) were prepared according to the previous work with grafting densities of 0.15 for PMCA and PMOXA brushes and 0.425 for the anchoring groups amine (NH2) and nitrodopamine (ND).^42–44^ The average degree of polymerization turned out to be 39 with both polymers, and the resulting molecular weights were 69 300 g mol^–1^ for the PMCA polymer and 48 300 g mol^–1^ for the PMOXA polymer.

### Synthesis and Coating of Gold NP

First, 117.45 mL of ultrapure water was stirred under reflux at 100 °C. In a separate glass vial, 5.6 mL of ultrapure water was stirred and mixed with 1.657 mL of a 510 mM sodium citrate solution (2.55 mmol in ultrapure water) and 250 μL of a 10 mM silver nitrate solution (0.4 mmol in ultrapure water), and the mixture heated to 35 °C. To the mixture was added 500 μL of a 0.25 M gold­(III) chloride trihydrate solution (0.13 mmol in ultrapure water). As soon as the mixture turned green (in around 3 min), it was transferred with a syringe to the refluxing boiling water. The resulting red mixture was stirred for 1.5 h at 100 °C. After cooling to room temperature, the mixture was separated into two batches (each 62.5 mL).

Then, 49.9 mg of PAcrAm-*g*-(PMCA, NH2, ND) (0.72 μmol) and 34.8 mg of PAcrAm-*g*-(PMOXA, NH2, ND) (0.72 μmol) were dissolved in 6.9 mL of HEPES I. The PMCA solution was added to the first batch of nanoparticles, and the PMOXA solution added to the second batch. Both batches were stirred for 19 h.

The coated nanoparticles were divided into 2 mL aliquots in Eppendorf tubes. The particles were separated from the reaction mixture by ultracentrifugation for 30 min at room temperature (RT) and 16 100 RCF. The supernatant was removed with a pipet, and the remaining precipitate dissolved in 1 mL of ultrapure water.

### Infrared Spectroscopy

The infrared spectra were acquired using a Nicolet Nexus 670 FT-IR spectrometer. Measurements were collected in transmission mode on KBr pellets, with each spectrum obtained by accumulating 20 scans at a resolution of 2 cm^–1^. An aliquot of 1 mL of the solution containing AuNP coated with PMCA and PMOXA was lyophilized. The lyophilized pellet was taken up with a drop of dichloromethane, and the mixture was placed on a KBr pellet. After the solvent had completely evaporated, the FT-IR spectrum of the sample was measured.

### DLS/ζ Potential Measurement

The hydrodynamic diameter was measured at 25 °C in ultrapure water, while the ζ potential was determined at 25 °C in a 10 mM PBS solution (pH 7.4) using a Malvern Zetasizer Nano-S equipped with a 633 nm HeNe laser and a Peltier temperature control system.

### Transmission Electron Microscopy (TEM) Microscopy

A FEI Tecnai G12 microscope operating at 100 kV was used to record the transmission electron micrographs, and the images were taken with an OSIS Veleta 4K camera. A drop of the nanoparticle suspension to be analyzed was deposited on standard grids, and the excess solution removed with absorbent paper.

### TGA

Thermogravimetric analysis (TGA) was performed using a Q5000 IR instrument from TA Instruments from 25 to 1000 °C under a continuous air flow. The temperature program included the heating of the samples at a rate of 10 °C/min to 100 °C, a constant temperature (100 °C) for 30 min, and then heating at a rate of 10 °C/min to 1000 °C.

The concentration of the suspensions of the PMCA- and PMOXA-PAcrAm-coated NP was determined with TGA, by dividing the residual mass of the samples after 30 min at 100 °C by the volume of the sample (0.1 mL for PMCA-NP and 0.3 mL for PMOXA-NP). The resulting concentrations were 0.20 mg/mL for PMCA-NP and 0.16 mg/mL for PMOXA-NP.

The final (>800 °C) percent weight loss (*W*) was used to determine the amount of polymers bound to the nanoparticles and the chain density parameters. The gold NP surface area (*A*_NP_) and volume (*V*_NP_) were calculated with eqs 1 and 2 assuming a spherical geometry.

1 $$A_{\mathrm{NP}}=4\pi {\left(\frac{d}{2}\right)}^{2}$$

2 $$V_{\mathrm{NP}}=\frac{4}{3}\pi {\left(\frac{d}{2}\right)}^{3}$$

these data allowed calculation of the number of polymer brushes per particle (*N*_brush_polymer_) with eq 3.

3 $$N_{\mathrm{brush\_polymer}}=\frac{gW\frac{\rho_{\mathrm{Au}}V_{\mathrm{NP}}}{100-W}N_{A}d_{\mathrm{brush\_polymer}}\times \mathrm{DP}}{M_{n\mathrm{\_polymer}}}$$

where ρ_Au_ is the density of gold (59 atoms nm^–3^), *N*_A_ Avogadro’s number, *M*_n_polymer_ the molecular weight of the polymers (69 300 g mol^–1^ for the PMCA polymer and 48 300 g mol^–1^ for the PMOXA polymer), DP the degree of polymerization (DP = 39 for both polymers), and *d*_brush_polymer_ the grafting density of the brushes (0.15 for both polymers).

The density of brushes on the surface of a gold NP (σ_brush_) was the calculated according to eq 4.

4 $$\sigma_{\mathrm{chain}}=\frac{N_{\mathrm{brush\_polymer}}}{A_{\mathrm{NP}}}$$

### Sodium Dodecyl Sulfate–Polyacrylamide Gel Electrophoresis (SDS–PAGE) Analysis

Frozen HS and PS were incubated for 5 min at 37 °C. Then, 180 μL of coated NP (PMCA-based polymer or PMOXA-based polymer) was mixed with 20 μL of phosphate-buffered saline (PBS buffer, 10-fold concentrated) and 300 μL of HS or PS to reach a NP concentration of 71 μg/mL for the PMCA-based polymer and 57 μg/mL for the PMOXA-based polymer. The particles were incubated at 37 °C for 20 min. The particles were centrifuged at 17 949 RCF for 40 min, and the supernatant was removed with a pipet. Afterward, the particles were washed twice: mixing with 1 mL of PBS (1-fold concentrated), centrifugation at 17 949 RCF for 40 min at 4 °C, and removal of the supernatant with a pipet.

The particles were mixed with 15 μL of loading buffer (62.5 mM Tris-HCl (pH 6.8), 2% (w/v) SDS, 25% (v/v) glycerol, 0.01% (w/v) bromophenol, and 1% (w/v) β-mercaptoethanol) and incubated at 95 °C for 5 min. The samples were added to wells of a 12% (w/v) polyacrylamide gel in an SDS–PAGE holder and subjected to 200 V and 15 mA until the running line is close to the end of the gel holder. Two different staining methods were then performed.

For silver staining, the gel was incubated while being shaken in a methanol/acetic acid solution (50% (v/v) methanol, 10% (v/v) acetic acid, and 40% (v/v) distilled water) for 30 min. Then the gel was incubated in a methanol/acetic acid solution (5% (v/v) methanol, 1% (v/v) acetic acid, and 94% (v/v) distilled water) for 5 min. The gel was then incubated in ultrapure water and shaken three times for 5 min, followed by a 90 s incubation with 0.2 mg/mL Na_2_S_2_O_3_. Three more washes of 30 s with ultrapure water were performed before incubation of the gel with a solution of 2 g/mL AgNO_3_ for 30 min under constant shaking. The gel is then revealed with an aqueous solution of 60 g/L Na_2_CO_3_, 0.05% (v/v) formaldehyde, and 4 μg/mL Na_2_S_2_O_3_ until the bands are clearly visible.

For Coomassie staining, the gel was stained with SimplyBlue SafeStain (Invitrogen) Coomassie stain following the manufacturer’s specifications.

### Cellular Assays

All studies involving human cells were conducted in accordance with the guidelines of the World Medical Association’s Declaration of Helsinki.

#### NF-kB-Luciferase Reporter Gene Assay

HEK293T cells were seeded at a density of 1.5 × 10^5^ cells per well in 24-well plates in 500 μL of high-glucose DMEM supplemented with 10% (v/v) heat-inactivated fetal calf serum (FCS), 2 mM MEM nonessential amino acids, 2 mM l-glutamine, 100 units/mL penicillin, and 100 μg/mL streptomycin. Transfection was done after 24 h, where 500 ng of DNA consisting of 100 ng of LPS receptor mix (with a 3:1:1 TLR4:MD2:CD14 ratio) and 400 ng of the NF-κB-luciferase reporter plasmid were diluted in 25 μL of Opti-MEM. Then, Lipofectamine 2000 (Invitrogen, Thermo Fisher Scientific) was diluted in Opti-MEM according to the manufacturer’s instructions and added to the DNA mixes in a ratio of 1:1. Then, 50 μL of the transfection mixes was then added to each well. After another 24 h, 500 μL of the same medium was added with the PMCA-based polymer-coated nanoparticles dissolved at a final concentration of 50 μg/mL, as well as medium with defined concentrations of LPS. Finally, after 24 h, the cells were lysed, and luciferase activity was measured after the addition of d-luciferin in white 96-well flat-bottom microtiter plates using a Tecan Infinite 200 Pro microplate reader.

#### Isolation of Blood-Derived Dendritic Cells

Peripheral blood mononuclear cells were isolated from fresh buffy coats, provided by the Hospital of Salzburg, by density gradient centrifugation with Histopaque-1077 (Sigma-Aldrich). Because national regulations do not require informed consent in the case of anonymous blood cells discarded after plasmapheresis (buffy coats), no additional approval by the local ethics committee is required. Then, dendritic cells were purified by magnetic depletion of CD19+ cells and positive selection of CD1c+ cells, using the Miltenyi Biotech CD1c (BDCA-1)+ Dendritic Cell Isolation Kit, according to the manufacturer’s specifications. Cells were ready immediately after the procedure.

#### Dendritic Cell Activation Assays

Dendritic cells isolated from peripheral blood were seeded in RPMI 1640, 10% (v/v) heat-inactivated FCS, 2 mM l-glutamine, and the PMCA-based polymer- and PMOXA-based polymer-coated nanoparticles added at a final concentration of 50 μg/mL. Lipopolysaccharide (LPS) was used in a concentration of 20 ng/mL as a positive control. Cells were incubated for 24 h at 37 °C. The following day, cells were recovered in microtubes and collected by centrifugation, and the supernatants were immediately stored at −80 °C.

Then, staining for flow cytometry was performed using CD86-FITC, HLA-DR-BV510, PD-L1-PE-Cy7, fixable viability dye eFluor 780, and CD1c-BV421, diluted according to the manufacturer’s specifications, and analyzed using a BD Biosciences FACSCanto flow cytometer after being fixed with 4% (v/v) paraformaldehyde. All antibodies were purchased from BD Biosciences.

For the quantification of TNFα, a sandwich enzyme-linked immunosorbent assay (ELISA) was performed, using a human TNFα ELISA development kit (ABTS) (Invitrogen) following the manufacturer’s specifications with respective previously frozen cell supernatants.

## Results and Discussion

Multicoordinating polymers were successfully used as NP coatings to improve their stability and ligand binding via multiple sites.^45^ Here, we use the recently introduced, multicoordinating PAcrAm polymer that combines electrostatically and covalently binding groups to create a robust coating.^43–47^ In particular, we selected two members of the PAcrAm family: PMCA-based polymer PAcrAm-*g*-(PMCA, NH2, ND), featuring multi-azide-containing polyoxazoline PMCA brushes and PMOXA-based polymer PAcrAm-*g*-(PMOXA, NH2, ND), featuring PMOXA homopolymer brushes (Chart 1). Both of these polymers feature the ability to bind inorganic surfaces exploiting both strong interactions and multivalency (Figure 1a).^3,45,49^ Indeed, nitrodopamine (ND) facilitates a robust dative–covalent binding to gold surfaces, primarily due to the chelation of gold by its catechol moiety. This form of binding closely mimics the natural adhesive proteins produced by mussels, which are renowned for their ability to adhere to various surfaces in aquatic environments. By leveraging this chelation mechanism, ND not only enhances the stability of the nanoparticle surface but also serves as a highly effective strategy for attachment of the polymer to metal surfaces.^44,50,51^ Additionally, amine groups are known to bind to gold surfaces more strongly than citrate, providing additional interaction points due to the polymer’s multivalent nature.^52^

**Chart 1:**
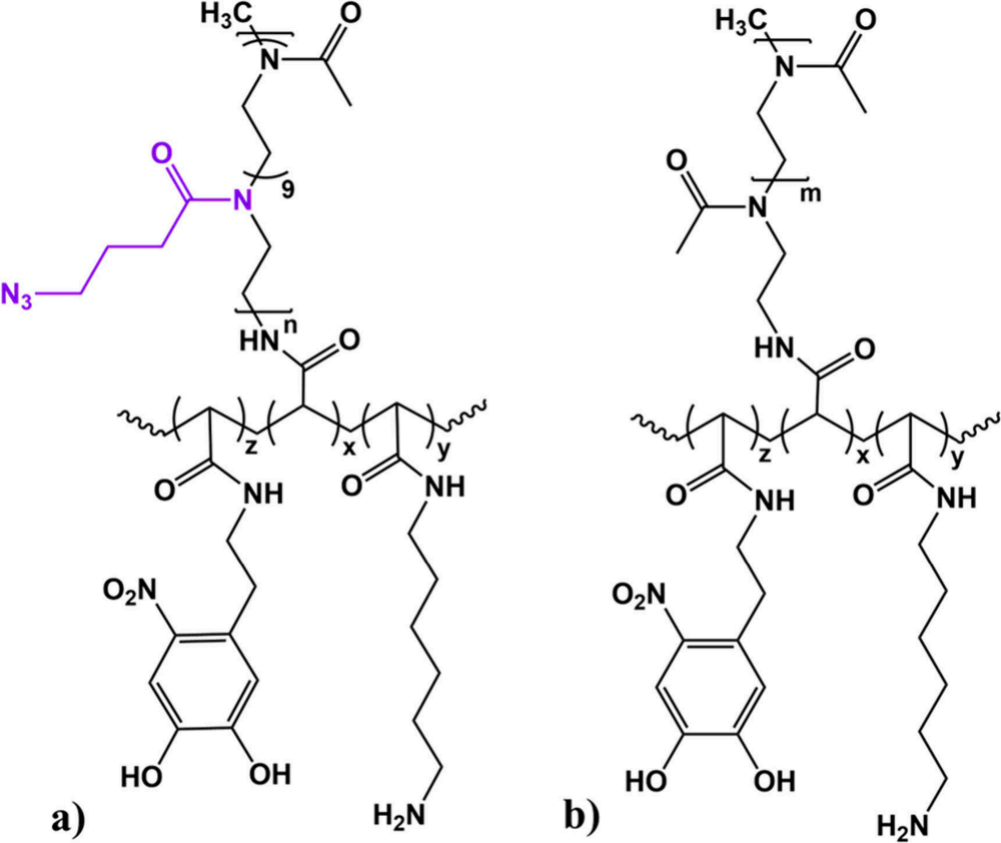
Structures of the Two Graft Copolymers^a^

a Conditions: (a) PAcrAm-*g*-(PMCA, NH2, ND) and (b) PAcrAm-*g*-(PMOXA, NH2, ND). The estimated values of the indexes are as follows: *m* ≈ 40, *n* ≈ 11, *x* ≈ 6, *y* ≈ 16, and *z* ≈ 16.

**Figure 1:**
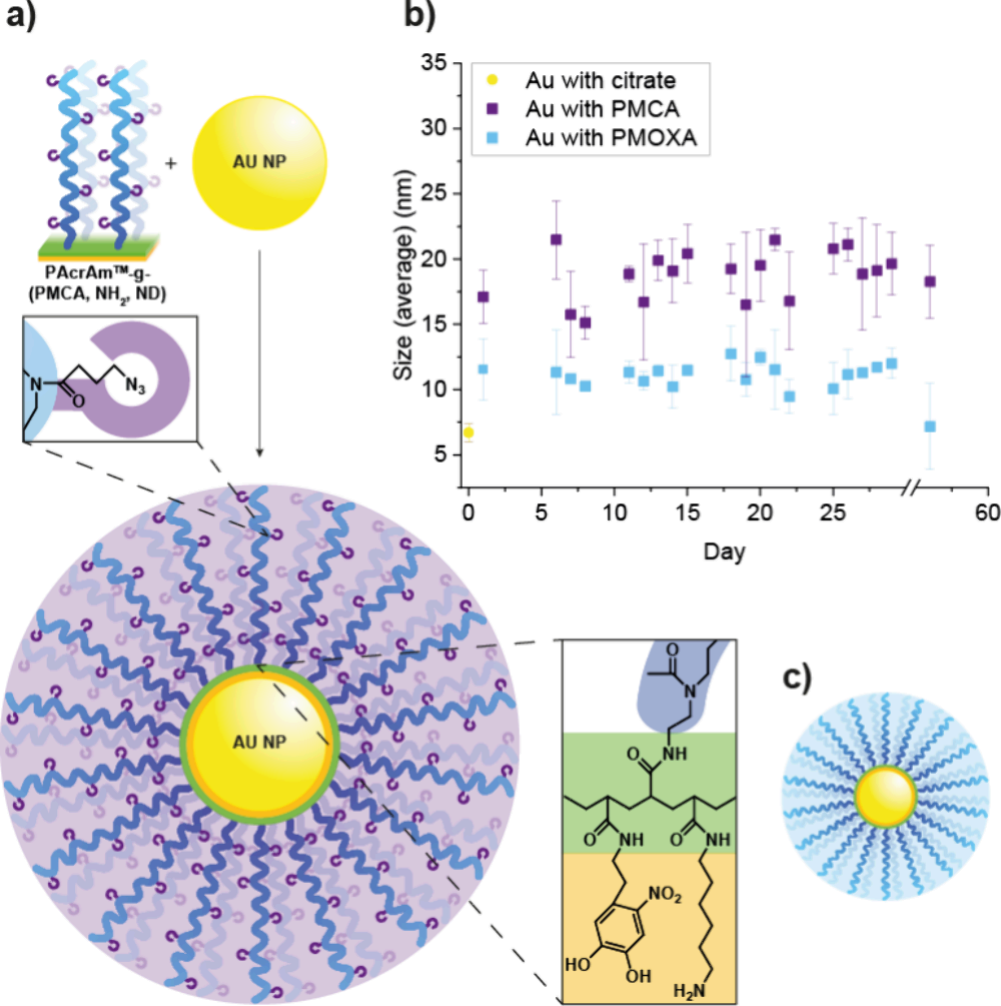
(a) Schematic representation of a Au-NP coated with PAcrAm-*g*-(PMCA, NH2, ND). Color code: PMCA, blue chain; azide, purple hook; PAcrAm backbone, green; anchoring groups, orange (ND on the left and NH2 on the right); Au-NP, yellow. (b) DLS measurement of the size of PMCA-based and PMOXA-based polymer-coated Au-NP over a time period of eight weeks (purple for PMCA and blue for PMOXA), with size at day 0 corresponding to that of the citrate-stabilized NP without a coating (yellow). (c) Schematic representation of Au-NP coated with PAcrAm-*g*-(PMOXA, NH2, ND).

Au-NP with diameter of about 8 nm were hence synthesized by a modification of the Turkevich method, wherein they were stabilized with a citrate layer (Figure S1).^48^ The citrate-stabilized Au-NP were mixed with the PMCA- or PMOXA-based polymers (5.4 μM) to replace the citrate with the polymers via ligand exchange, followed by purification via centrifugation and resuspension cycles. PAcrAm-coated NP prepared in this way were then subjected to extensive investigations.

### Nanoparticle Coating Characterization

An incomplete coating can influence NP cytotoxicity, uptake, and biodistribution.^53,54^ To assess the effectiveness of the polymer coatings, we characterized the Au-NP with FT-IR, dynamic light scattering (DLS), ζ potential analysis, and TEM. Further information was obtained by TGA and will be discussed below.

Purified nanoparticles were lyophilized and analyzed by transmission FT-IR (see Figure S6). Besides a residual water band, all of the other signals detected were identical to those of the pristine PAcrAM-PMCA and PMOXA polymers.^42^ In particular, the characteristic asymmetric stretching band of the azide group (N_3_) was detected at 2097 cm^–1^ only in the spectrum of the PMCA-coated nanoparticles. The typical signals of citrate adsorbed on gold at 1620 and 1390 cm^–1^ could not be detected,^63–67^ but their masking by the strong polyoxazoline bands at 1640 and 1415 cm^–1^ cannot be excluded. At any rate, FT-IR spectra indicate the almost quantitative or prevalent substitution of citrate with the two polymers, as confirmed by the results of ζ potential measurements.

DLS measurements provided the hydrodynamic diameter, *D*_h_, of the citrate-stabilized (6.7 ± 0.7 nm) and PAcrAM-coated Au-NP (17.1 ± 2.0 nm for PMCA and 11.3 ± 2.4 nm for PMOXA) (Table 1). The diameter measured for the citrate-stabilized nanoparticles agrees with the core size, *D*_core_, measured by TEM (Table 1). In the case of the PAcrAm-coated Au-NP, the size increase observed indicated that the polymers successfully grafted to the particles’ surface in both cases, forming a coating layer on them.

**Table 1:** Diameters (*D*_h_ and *D*_core_), ζ Potentials (ζ), and Polydispersity Indices (PDI) of Citrate-Stabilized Nanoparticles and PMCA-Based and PMOXA-Based Polymer-Coated Nanoparticles in Water at 21°C

nanoparticles	*D*_core_ (nm)^a^	*D*_h_ (nm)^b^	ζ (eV)	PDI
Au-NP-citrate^b^	8.7 ± 1.5	6.7 ± 0.7	–67.3 ± 4.0	0.586
Au-NP-PMCA	9.0 ± 1.5	17.1 ± 2.0	–0.3 ± 0.2	0.376
Au-NP-PMOXA	8.6 ± 1.6	11.3 ± 2.4	–6.35 ± 1.0	0.453

a Measured by TEM.

b Number-weighted size distribution measured with DLS.

The larger *D*_h_ for the NP coated with the PMCA-based polymer was attributed to the different sizes of the PMCA and PMOXA brushes in the two coating copolymers. The average molecular weight for PMCA was indeed 10 500 g mol^–1^ compared to that of PMOXA, which was 6900 g mol^–1^. Increments of the coating thicknesses by 6–7 nm, similar to those here observed, were previously observed for quantum dots coated with a multicoordinating polymer consisting of a poly­(isobutylene-*alt*-maleic anhydride) (PIMA) backbone with PEG brushes.^55^ Other studies on nanoparticles coated with a similarly sized polyoxazoline chains reported coating thicknesses between 5 and 20 nm.^25,56–58^

Polydispersity indices (PDI) of the three Au-NP batches, measured by DLS, were quite high, suggesting aggregation or a wide size distribution.^59–62^ However, PDI values for the polymer-coated Au-NP were smaller than those of the citrate-coated Au-NP (Table 1), indicating that citrate displacement did not produce a relevant aggregation of the particles.

This conclusion was further supported by TEM. First, the average diameters of the gold cores around 8.8 nm were determined for all of the samples (Table S1). This indicates that no particle etching or secondary growth occurs during the coating exchange. Second, the TEM images of the polymer-coated nanoparticles evidenced the complete absence of aggregation (Figure S2), confirming a good stabilization of the particles by the polymer coating.

ζ potential measurements were then performed to obtain information about the degree of replacement of the citrate layer by the polymers. Literature ζ values for citrate-coated Au-NP in water range from −50 to −35 eV, as result of the negative charge of the coating molecules.^56,63–67^ Accordingly, the measured ζ for our citrate-coated Au-NP was −67 ± 4.0 eV. Most importantly, the ζ values measured for the polymer-coated Au-NP are close to zero (−0.3 ± 0.2 and −6.35 ± 1.0 eV for the PMCA- and PMOXA-coated particles, respectively), confirming the complete or almost complete replacement of the negatively charged citrate ions.^56^ This also indicates that the colloidal stability of the coated NP is indeed due to the steric repulsion by the neutral organic coating and not induced by charge repulsion.

Eventually, to investigate the long-term stability of the polymer-coated nanoparticles, we monitored their hydrodynamic diameter for 2 months using DLS (Figure 1b). For both batches, the NP size remained the same, confirming an excellent stabilization by the coating for at least two months. These results were confirmed by TEM analysis after four weeks that showed the absence of detectable nanoparticle aggregation (Figure S3).

### Brush Polymer Density

As mentioned in the Introduction, the nonspecific adsorption of proteins on the surface of NP remains a challenge for NP-based therapeutics.^8,13,14^ The formation of a protein corona can significantly influence the fate of the NP in the body by influencing the biodistribution, cellular uptake, and cytotoxicity.^8,10,13,14^ In addition, opsonization can trigger macrophage capture. To maximize their ability to suppress nonspecific interaction with proteins, the hydrophilic nonfouling polymers coating the NP should have a brush-like conformation.^8,14^ This conformation is reached at high chain surface densities (σ_chain_) and results in the elongation of the polymer chains due to their close packing. On the other hand, at low σ_chain_ values, the chains can relax and adopt mushroom- or pancake-like structures that are less resistant to protein absorption.^8,68–70^ Dahal et al. postulated the transition from a mushroom-like conformation to a brush-like conformation on NP to happen when the σ_chain_ increases above a critical value (σ_chain_^*^) determined by eq 5:^71^

5 $$\sigma_{\mathrm{chain}}^{*}=\left(\frac{1}{R_{G}}+\frac{1}{R}\right)$$

where *R*_G_ is the polymer radius of gyration and *R* is the NP core radius determined by TEM. *R*_G_ values of the polymers used here were 3.69 and 2.88 nm for the PMCA- and PMOXA-based derivatives, respectively, calculated with the Fox–Flory formula (eqs S1–S3).^42^

The amount of polymer bound to the Au-NP was determined by TGA in air (Figures S4 and S5). In these experiments, the total weight loss (*W*) observed upon heating above 100 °C corresponds to the decomposition of the organic component while the residual weight corresponds to the metal component. The obtained data allowed calculation of the number of polymer chains (PMCA or PMOXA) per NP (*N*_brush_polymer_), the chain density (σ_chain_), and the chain footprint (Table 2).^25,72^

**Table 2:** TGA Weight Losses (*W* (percent)), Numbers of Brush-Forming Polymers per NP (*N*_brush_polymer_), Coated Au-NP Chain Densities (σ_chain_ (inverse square nanometers)), Chain Footprints (square nanometers), and Critical Values for Brush Formation (σ_chain_^*^, inverse square nanometers)

nanoparticles	weight loss *W* (%)	*N*_brush_polymer_	σ_chain_ (nm^–2^)	footprint (nm^2^)	σ_chain_^*^ (nm^–2^)
Au-NP-PMCA	49.9	238	1.26	0.79	0.28
Au-NP-PMOXA	37.3	204	1.08	0.92	0.37

Table 2 reveals that a greater weight loss *W* was observed for NP coated with the PMCA-based polymer than for the PMOXA-based polymer. However, because of the different molecular weights of the two polymers (10 500 g mol^–1^ for PMCA and 6900 g mol^–1^ form PMOXA), this resulted in similar surface grafting densities (σ_chain_) for the two coatings.

Compared to flat gold surfaces, the σ_chain_ values increased from 0.13 to 1.26 chains/nm^2^ for the PMCA-based polymer and from 0.21 to 1.08 chains/nm^2^ for the PMOXA-based polymer.^42^ Similar results were obtained in previous work using poly­(2-ethyl-2-oxazoline) (PEOXA) with a degree of polymerization comparable to that of PMCA assembled on flat surfaces and Au-NP with comparable sizes (<10 nm).^25,73^ In that case, measured chain density σ_chain_ increased from 0.1 chain/nm^2^ on planar surfaces to 1.2 chains/nm^2^. The increase in σ_chain_ from planar surfaces to NP surfaces was attributed to the curvature of the Au-NP surface,^73,74^ which allows decreased polymer crowding far from the surface and therefore denser packing at the surface.^71,75^ The most important observation, however, is that for both Au-NP batches σ_chain_ exceeded the critical value σ_chain_^*^ (Table 2), supporting a brush-like conformation as drawn in the schematic representation (Chart 1a).

### Protein Adhesion

Subsequently, we investigated the formation of the protein corona on the surface of the coated NP to assess how the coatings modulate the interactions with serum proteins. NP were incubated for 20 min at 37 °C with 60% (v/v) human serum (HS) or pig serum (PS). No particle aggregation was observed under these conditions. The corona-coated NP were subsequently isolated and washed by centrifugation cycles. The adhered proteins (on the coated NP as well as on the container wall) were then detached and characterized using SDS–PAGE (Figure 2c,d).

**Figure 2:**
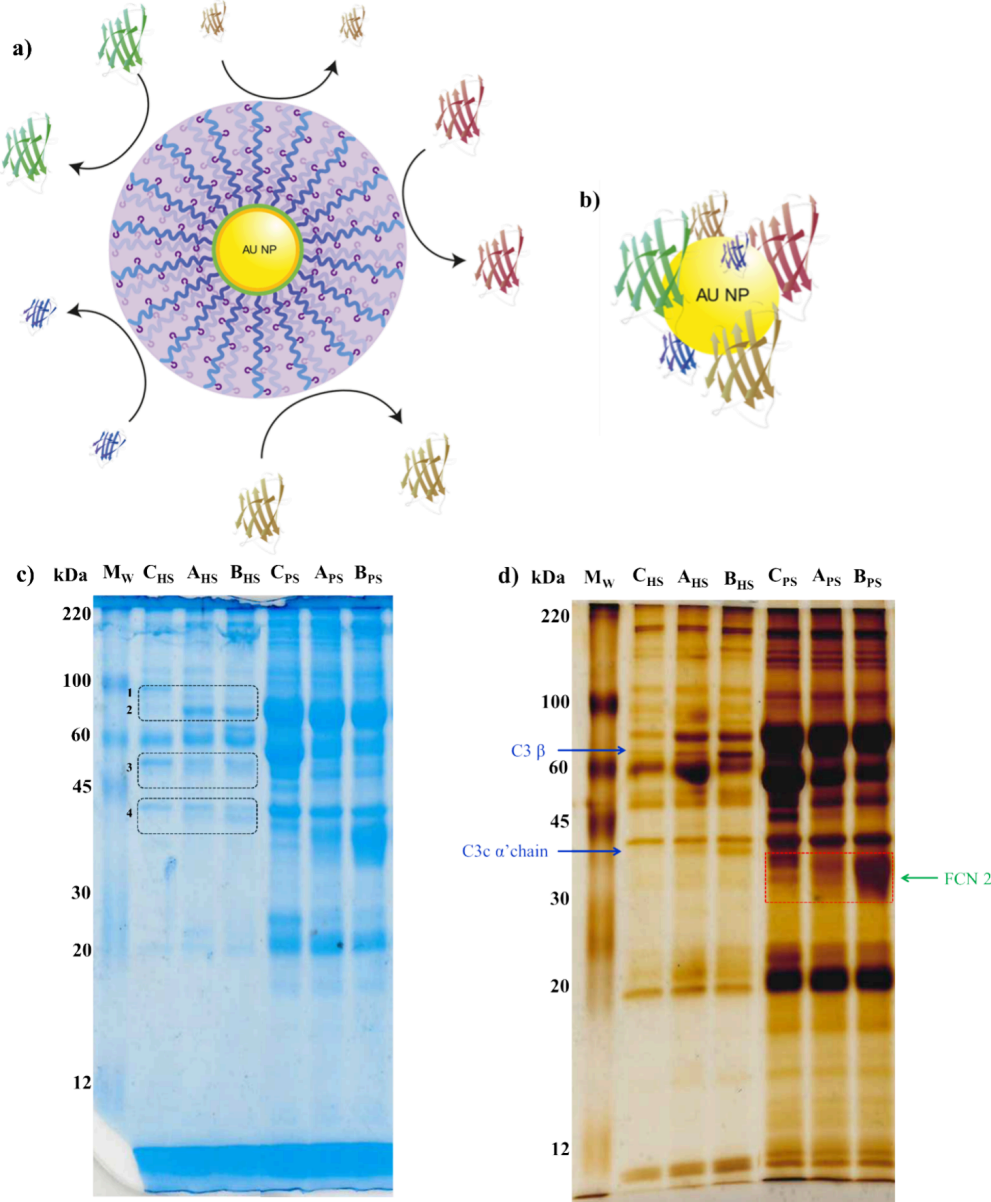
Protein adsorption analysis of polymer-coated Au-NP. (a) Schematic illustration of the suppression of nonspecific protein adhesion on Au-NP coated with the PMCA-based polymer. (b) Formation of a protein corona around uncoated Au-NP due to uncontrolled adsorption. (c) SDS–PAGE of proteins recovered from Au-NP after exposure to a serum protein (HS or PS). A dashed square highlights distinct bands corresponding to adsorbed proteins at 80 (1), 70 (2), 50 (3), and 39 kDa (4). (d) Silver-stained SDS–PAGE of the same samples, with a red dashed box emphasizing a prominent band at ∼38 kDa, indicating a high-affinity protein interaction in PS, corresponding to pig FCN 2 (green arrowhead). Blue arrowheads indicate human C3β (70 kDa) and C3c a′ chain fr.2 (39 kDa). Labeling of the individual lanes: Mw, molecular weight calibration proteins; C_HS_, control HS, no NP, only from nonspecific adsorption of proteins from container walls; A_HS_, PMCA-coated Au-NP exposed to HS; B_HS_, PMOXA-coated Au-NP exposed to HS; C_PS_, control PS, no NP, only from nonspecific adsorption of proteins from container walls; A_PS_, PMCA-coated Au-NP exposed to PS; B_PS_, PMOXA-coated Au-NP exposed to PS.

Noticeably, most of the proteins detected in the NP coronas (columns A_HS_, B_HS_, A_PS_, and B_PS_ in Figure 2c) were also present in the control runs (lanes C_HS_ and C_PS_), which corresponded to serum samples not containing NP and treated in the same way as NP-containing ones. The proteins found in these lanes (C_HS_ and C_PS_) correspond to the background residual proteins not fully removed with the washing cycles, possibly because of their adhesion to the walls of the tubes. The observation that the control and two NP lanes feature similar patterns and band intensities suggested a low level of protein adsorption by the coated nanoparticles and confirmed the general antifouling nature of the polyoxazolines.

Nevertheless, a careful inspection revealed the presence of a few additional proteins in the NP coronas, and interesting differences, albeit subtle, between the two coatings. First, densitometric quantification indicated a lower level of protein absorption on PMCA-coated NP than on PMOXA-coated ones (Table S2). The main HS NP corona bands corresponded to proteins with molecular weights of 80, 70, 50, and 39 kDa. The bands at 80 and 50 kDa could not be identified, and they were present in similar amounts on both coated NP. On the other hand, the two bands at 70 and 39 kDa (blue arrows in panels c and d of Figure 3) have been reproducibly identified as major components of PMOXA-coated NP corona^31,32^ and correspond to the C3 β and C3c α′ chain fr.2 subunits of the C3 protein. The presence of C3c α′ chain fr.2 indicates complement activation and the formation of strong opsonin iC3b. Remarkably, these two C3-derived bands were less intense in PMCA nanoparticles (lane A_HS_) than in PMOXA nanoparticles (lane B_HS_), suggesting a weaker complement activation promoted by the PMCA coating with respect to PMOXA.

**Figure 3:**
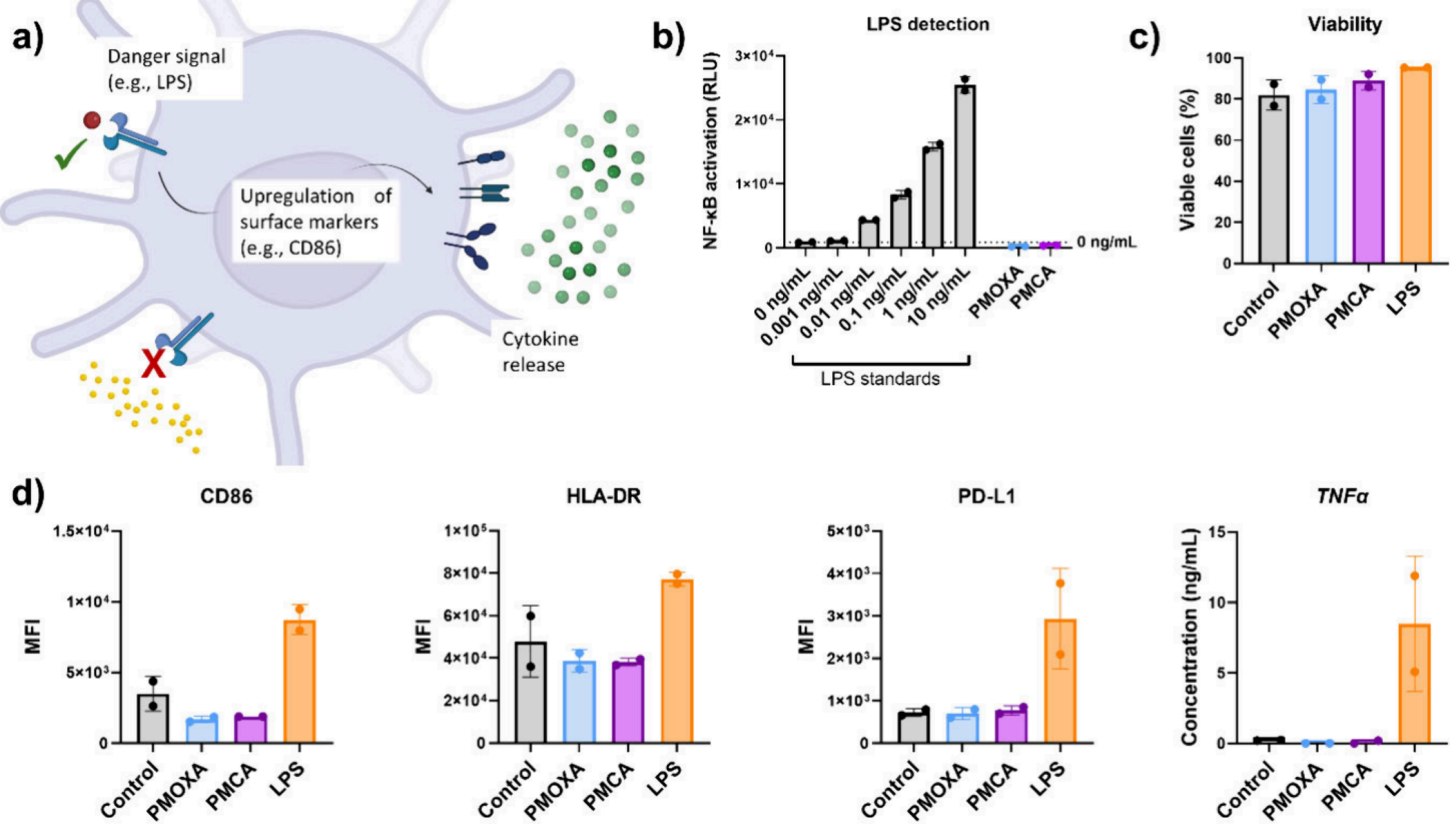
PMOXA- and PMCA-coated particles do not trigger dendritic cell activation. (a) Schematic representation of the activation process. Activation via pattern recognition receptors induces dendritic cell activation. This makes DCs suitable for antigen presentation, while simultaneously triggering an inflammatory reaction. Typical signs of activation include the upregulation of certain surface markers, such as CD86, and cytokine release. (b) Particles were tested for the presence of lipopolysaccharide (LPS) using a NF-kB reporter gene assay in TLR4-transfected HEK293T cells. LPS in the concentration range of 0–10 ng/mL was used as a positive control. Each point in panels c and d corresponds to a different donor. RLU = relative light units; MFI = median fluorescence intensity.

Recently, the protein ficolin-2 (FCN2) was found to strongly bind PMOXA-coated NP in pig serum, where it is present at high concentrations.^32^ This protein is a pattern recognition receptor of innate immunity, which recognizes *N*-acetyl units as those present in PMOXA, leading to NP opsonization.^32^ FCN2 binds less intensely to PMOXA-coated NP incubated in HS, since its concentration in human serum is reduced compared to that in pig serum, and in addition, its presence is more dependent on the serum donor. Hence, since FCN2 binding is a good model to test the ability of NP coats to evade the innate immune system, we decided to investigate in detail the presence of this protein in the NP coronas formed in HS and also in PS.

As expected, in human serum it was not possible to detect the FCN2 band at 38 kDa (Figure 3c,d). As mentioned, this could be due to low levels of this protein in the serum used. However, when both NP were incubated in pig serum, where FCN2 is more abundant, the band corresponding to this protein (green arrow in Figure 3d) was clearly detected as one of the major corona components. Remarkably, we observed also in the case of FCN2 weakened binding to PMCA coatings compared to PMOXA coatings (Table S10).

In summary, SDS–PAGE analysis indicated that few proteins can attach to the surface of the coated NP, confirming the resistance of the coatings to nonspecific protein adhesion previously observed for both polymers on flat surfaces.^42^ Even more interesting is the fact that these preliminary results suggested a reduction in complement activation in the case of PMCA coatings and a decrease in the ability of the specific pattern recognition molecule FCN2 to bind to PMCA coatings with respect of PMOXA coatings. This evidence suggests an enhanced propensity of PMCA coats to evade potential opsonizing and complement activating serum agonists.

### Dendritic Cell Activation

Besides preventing protein corona formation, a key point in designing a stealth nanosystem is avoiding recognition and subsequent activation of the immune response. Having verified that protein adsorption is low, we then investigated if the NP induced any cellular response in human CD1c+ dendritic cells, which are among the main mediators of induced immune responses. DC activation is initiated upon recognition of foreign or dangerous material by pattern recognition receptors, inducing phenotypical changes that can initiate a strong immune response (Figure 3a).^76,77^

An important caveat when CD1c+ dendritic cells are used in experiments is that they are extremely sensitive to ubiquitously present endotoxins, i.e., LPS, detecting concentrations as low as 20 pg/mL.^78^ To ensure that the potential effects induced by NP in these cells were not a consequence of residual endotoxin contamination in the formulations, we used a TLR4-NF-κB reporter gene assay, which confirmed the absence of LPS in all of the tested samples (Figure 3b).

Then, to determine if the particles induced DC activation, we monitored the surface expression of CD86, HLA-DR, and PD-L1 and the release of TNFα 24 h after treatment with the Au-NP. As shown in Figure 3d, neither PMOXA- nor PMCA-coated particles led to upregulation of the activation markers (CD86, HLA-DR, and PD-L1) or to the release of TNFα, and thus, neither triggered dendritic cell activation in contrast to the LPS used as a positive control. This indicates that the two coatings are not recognized as pathogen- or damage-associated molecular patterns and should not trigger an immune response, reducing the likelihood of immune-related side effects.

Additionally, the nanoparticles had no impact on cell viability (Figure 3c). In summary, the PMCA particles represent a highly tunable platform with the potential to be used for a wide range of applications, from drug delivery to imaging.

## Conclusions

The coating of gold nanoparticles with the multi-azide polymer PAcrAm-*g*-(PMCA, NH2, ND) is demonstrated to be straightforward and provides effective stabilization of the nanoparticles for at least two months. This extended stability is crucial for maintaining the integrity and functionality of the nanoparticles during potential biomedical applications. The brush-like conformation of the PMCA polymer significantly suppresses nonspecific protein adhesion, which is pivotal for enhancing the circulation time of nanoparticles within the body. Notably, the PMCA coating shows a pronounced ability to apparently suppress opsonization, which could offer significant advantages for nanomedicine applications.

The speculation that such an effect is due to the presence of the azidopropyl moieties, notwithstanding their low abundance, in the PAcrAm brushes is intriguing. Indeed, the irregular occurrence of nonrecognized moieties in the polymer sequence, or even their possible concentration in the terminal part of the polyoxazoline chains, could decrease the affinity of FCN2, which is evolutionarily optimized to recognize specific patterns, such as regular *N*-acetyl repetition.^31,32^ However, more information will be needed to confirm this hypothesis.

Another relevant confirmation of the biocompatibility of the polymer coating is that neither of the particles triggers dendritic cell activation or has any effect on their viability, decreasing the probability of immune system-related side effects and clearance by the mononuclear phagocyte system.

Ultimately, the versatile nature of the bio-orthogonal azide–alkyne reaction will facilitate the straightforward incorporation of various biomolecules onto the nanoparticle surface. When combined with the nonfouling properties of the polymer and low immunogenicity, which suggests prolonged circulation times, the coated nanoparticles can be modified for a wide range of biomedical applications, including targeted drug delivery, diagnostics, and vaccine development. This versatility positions PAcrAm-*g*-(PMCA, NH2, ND) as a promising candidate for advancing nanotechnology in healthcare.^79,80^

## Supplementary Material



## Notes

Experiments (DLS and TEM) for the evaluation of the shelf stability of coated nanoparticles, experiments (TGA) and calculations for the estimation of the coating density of the polymer brushes on the nanoparticles, FT-IR analysis of the coated nanoparticles, and densitometric quantification of the corona proteins from SDS–PAGE analyses (PDF)
