# Supplementary material for: Conjugable, Antifouling, and Non-immunogenic Coatings for Gold Nanoparticles by Multivalent Grafting of Azide-Bearing Polyoxazoline Brushes
Source: Langmuir. 2026 Jan 28;42(5):3800–10. doi: 10.1021/acs.langmuir.5c04094 (PMC12895522; doi:10.1021/acs.langmuir.5c04094)
Supplement: Supplementary file 1 [file la5c04094_si_001.pdf]

Supporting Information

Conjugable, antifouling and non-immunogenic coatings for gold nanoparticles by multivalent grafting of azide-bearing polyoxazoline brushes

Tobias Komsthöft,[1,2]‡ Michele do Nascimento Tomaz,[3]‡ Pedro R. M. Veloso,[4] Ana R. S. Ribeiro,[5] Lucca Trachsel,[1,6] Jutta Horejs-Höck,[5] Mark W. Tibbitt,[2] Samuele Tosatti,[1] Emanuele Papini,[4,] Stefan Zürcher,[1,*] Fabrizio Mancin[3,*]*

[1] SuSoS AG, Lagerstrasse 14, 8400 Dübendorf, Switzerland
[2] Macromolecular Engineering Laboratory, Department of Mechanical and Process Engineering, ETH Zurich, 8092 Zurich, Switzerland
[3] Department of Chemical Sciences, University of Padova, Via F. Marzolo 1, 35121 Padova, Italy
[4] Department of Biomedical Sciences, University of Padova, Via U. Bassi 58/b, 35121 Padova, Italy
[5] Department of Biosciences and Medical Biology, Paris Lodron University of Salzburg, Salzburg, Austria
[6] Department of Chemistry, Carnegie Mellon University, 4400 Fifth Avenue, Pittsburgh, Pennsylvania 15213, United States

E-mail: emanuele.papini@unipd.it, szuercher.board@susos.com, fabrizio.mancin@unipd.it

Contents:

1. Shelf Stability of the Polymer Coated Gold Nanoparticles.. 2

2. Estimation of the coating density..5

2.1 TGA analysis of the coated nanoparticles...5

2.2 Calculation of the polymers' radius of gyration..6

3. Densitometric relative quantification of FCN2 band..6

4. References..6

1. Shelf Stability of the Polymer Coated Gold Nanoparticles

Table S1: Time-dependent average size of coated gold nanoparticles measured by TEM over 27 days, showing no significant changes in size.

	Size (nm)	Standard deviation
Au NP-citrate after 0 days	8,93	±1,88
Au NP-PMCA after 6 days	9,04	±1,54
Au NP-PMCA after 14 days	9,57	±1,96
Au NP-PMCA after 19 days	8,48	±1,55
Au NP-PMCA after 27 days	8,66	±1,95
Au-NP-PMOXA after 6 days	8,56	±1,58
Au-NP-PMOXA after 14 days	8,43	±1,51
Au-NP-PMOXA after 19 days	8,29	±1,55
Au-NP-PMOXA after 27 days	9,01	±2,11

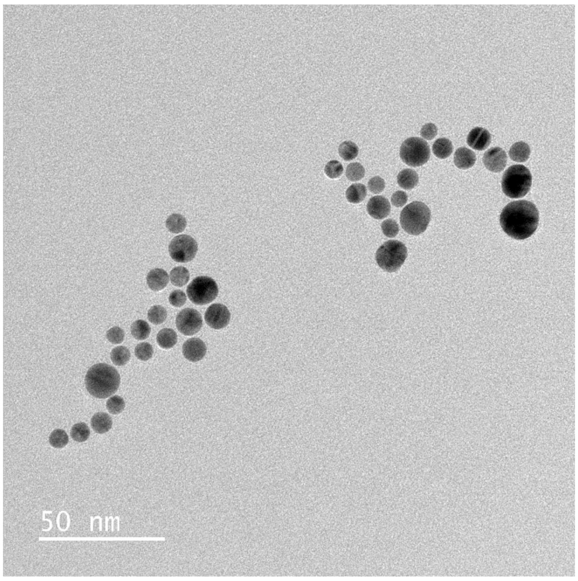

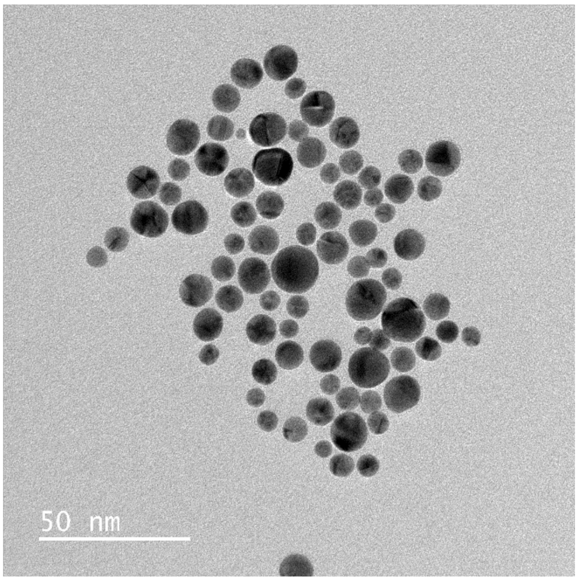

Figure S1: Gold nanoparticles stabilized with citrate.

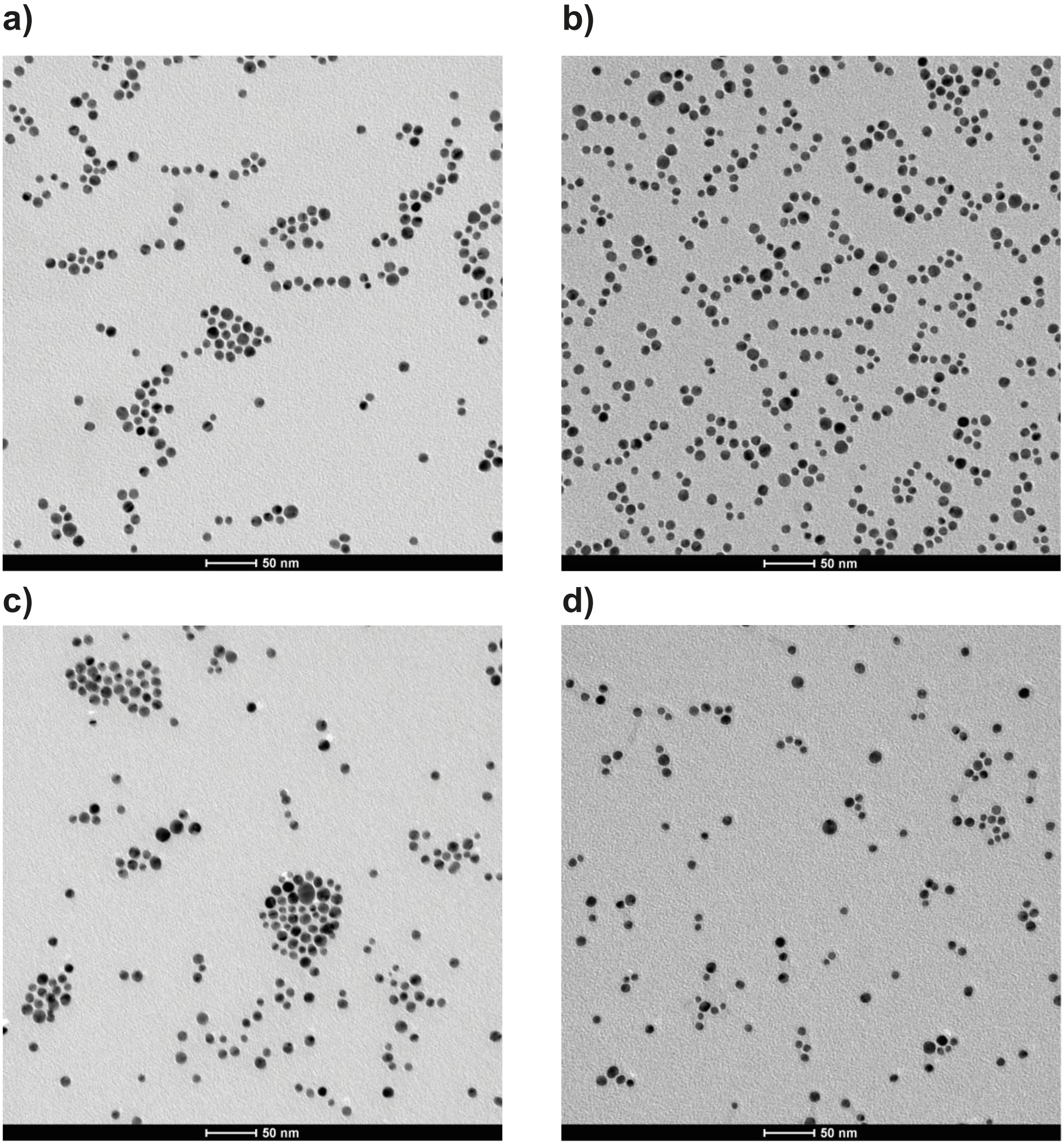

Figure S2: PMCA-based polymer coated NP after a) 6 days and c) 14 days; PMOXA-based polymer coated NP after b) 6 days and d) 14 days.

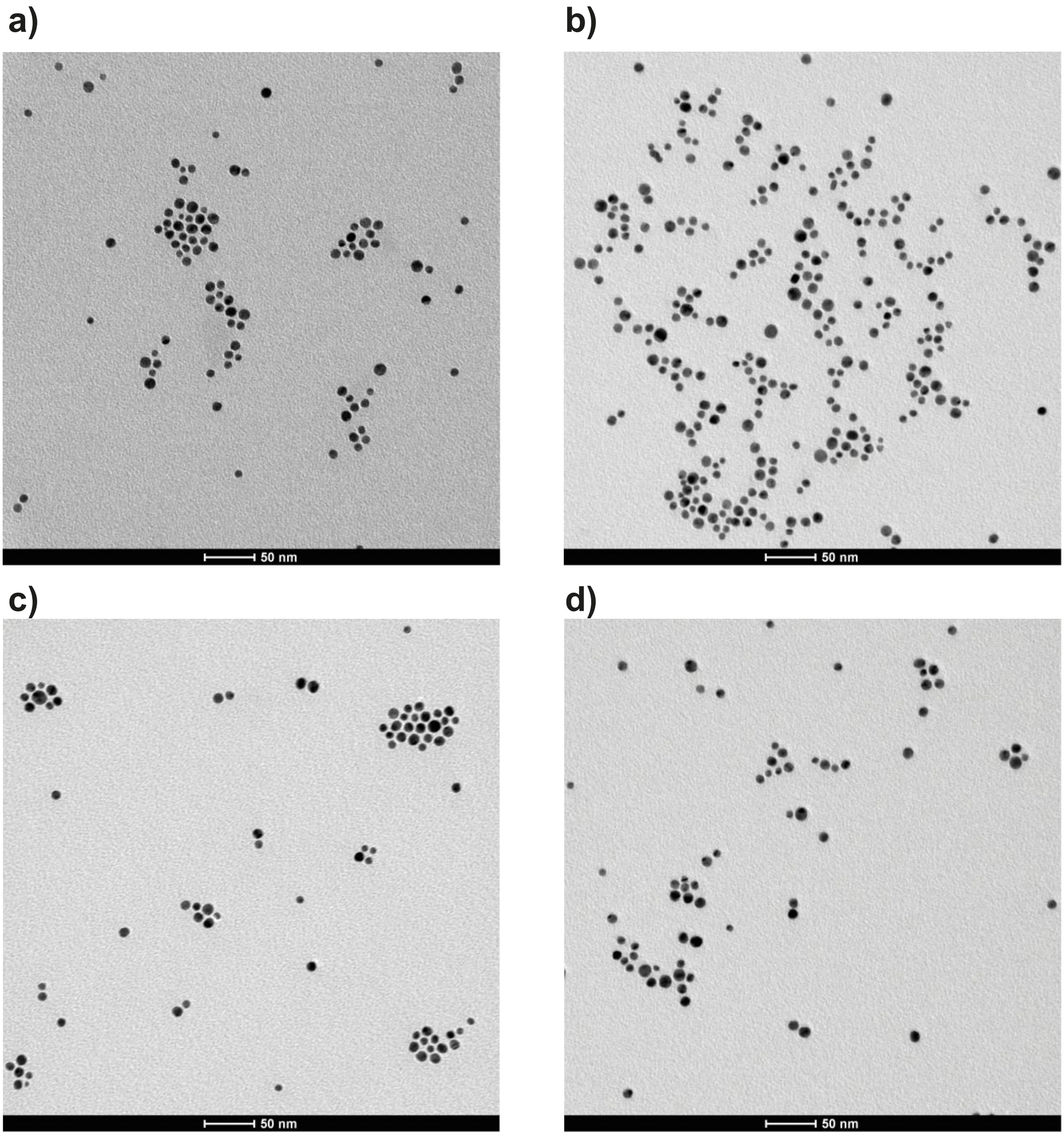

Figure S3: PMCA-based polymer coated NP after a) 19 days and c) 27 days; PMOXA-based polymer coated NP after b) 19 days and d) 27 days.

2. Estimation of the coating density

2.1 TGA analysis of the coated nanoparticles

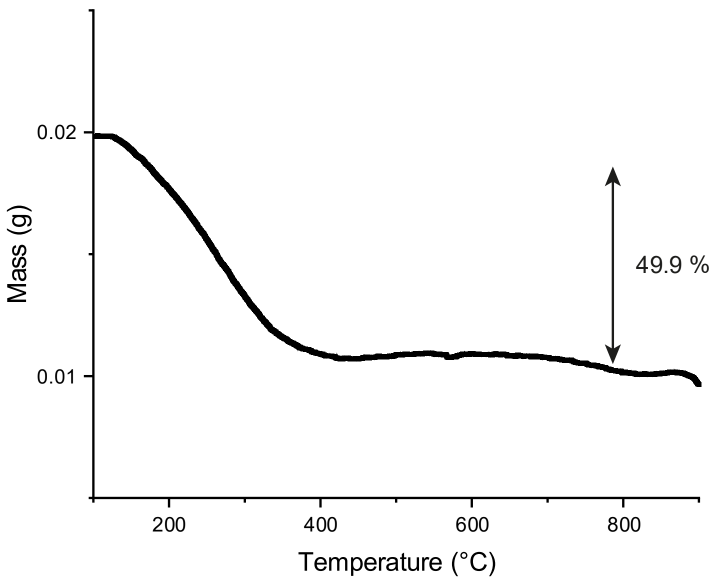

Figure S4: TGA thermogram (in air) of PMCA-based polymer coated NP. The resulting weight loss, due to the thermolysis of the polymer coating, is 49.9% of the total mass.

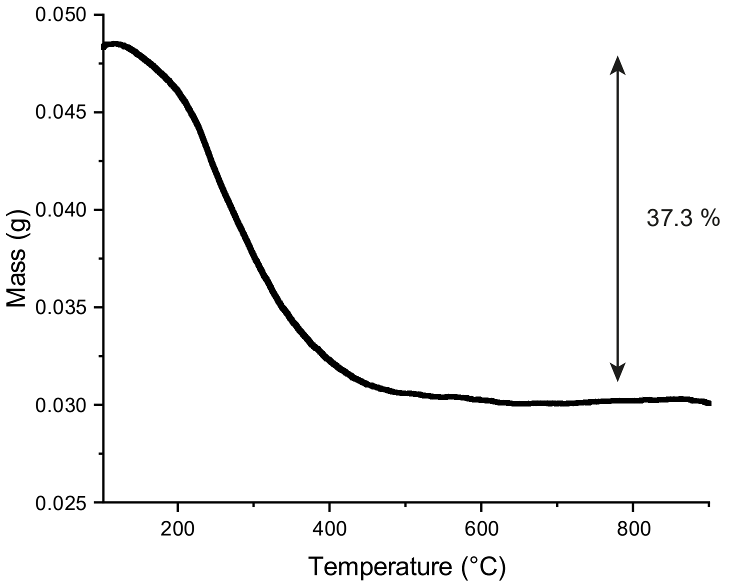

Figure S5: TGA thermogram (in air) of the PMOXA-based polymer coated NP. The resulting weight loss, due to the thermolysis of the polymer coating, is 49.9% of the total mass.

2.2. Calculation of the polymers' radius of gyration

The radius of gyration RG of the polymers were calculated with **Equation S1**:

$$R_G = \left(\frac{M_n \cdot [\eta]}{\Phi \cdot 6^{\frac{3}{2}}}\right)^{\frac{1}{3}} \quad \text{(S1)}$$

The intrinsic viscosity [η] of PMOXA can further be estimated in water at 20°C with the molecular weights of PMCA and PMOXA (10521 g mol^{-1} and 6925 g mol^{-1} respectively) **Equation S2**:[1,2]

$$[\eta] = 0.015 \cdot M_n^{0.77} \quad \text{(S2)}$$

Although PMCA is supposedly bulkier due to the APOXA monomer, we used Equation S2 as an estimation for PMCA's η. The intrinsic viscosity for PEG can be calculated with **Equation S3**:[3,4]

$$[\eta] = (0.02 + 2.4 \cdot 10^{-4} \cdot M_n^{0.73}) \cdot 100 \quad \text{(S3)}$$

3. Densitometric relative quantification of FCN2 band

ImageJ was used for densitometric analysis of the silver stained SDS-PAGE gels. For the lanes of interest, the selection tool was used to select the band present at approximately 35 kDa. Then, from the respective plot, the area of the peak was obtained, and the relative amounts were calculated by dividing each value for the area of the control sample (CPS).

Table S2: Area and relative amounts of pig ficolin-2 (FCN2), obtained by densitometric quantification of the bands in the SDS-PAGE gels reported in **Figure 2d**. (approx. 35 kDa).

Lane	FCN2 band area (a.u.)	Relative amount vs. C_{PS}
**C_{PS}**	1340.97	1.00
**A_{PS} (AuNP-PMCA)**	10367.85	7.73
**B_{PS} (Au NP-PMOXA)**	26061.38	19.44

4. REFERENCES

(1) Komsthöft, T.; Bartalucci, N.; Tibbitt, M. W.; Tosatti, S.; Zürcher, S. Non-Fouling Multi-Azide Polyoxazoline Brush-co-Polymers for Sensing Applications. Adv. Mater. Interfaces 2024, 11 (35), 2400322. https://doi.org/10.1002/admi.202400322.

(2) Gubarev, A. S.; Lezov, A. A.; Podsevalnikova, A. N.; Mikusheva, N. G.; Fetin, P. A.; Zorin, I. M.; Aseyev, V. O.; Sedlacek, O.; Hoogenboom, R.; Tsvetkov, N. V. Conformational Parameters and Hydrodynamic Behavior of Poly(2-Methyl-2-Oxazoline) in a Broad Molar Mass Range. Polymers 2023, 15 (3), 623. https://doi.org/10.3390/polym15030623.

(3) Kirinčič, S.; Klofutar, C. Viscosity of Aqueous Solutions of Poly(Ethylene Glycol)s at 298.15 K. Fluid Phase Equilibria 1999, 155 (2), 311–325. https://doi.org/10.1016/S0378-3812(99)00005-9.

(4) Ring, W.; Cantow, H.-J.; Holtrup, W. Molekulargewichte und Molekulargewichtsverteilungen von Polyäthylenoxiden. Eur. Polym. J. 1966, 2 (2), 151–162. https://doi.org/10.1016/0014-3057(66)90070-X.
